# MLIP genotype as a predictor of pharmacological response in primary open-angle glaucoma and ocular hypertension

**DOI:** 10.1038/s41598-020-80954-2

**Published:** 2021-01-15

**Authors:** María I. Canut, Olaya Villa, Bachar Kudsieh, Heidi Mattlin, Isabel Banchs, Juan R. González, Lluís Armengol, Ricardo P. Casaroli-Marano

**Affiliations:** 1 Centro de Oftalmología Barraquer, Instituto Universitario Barraquer (UAB), Barcelona, Spain; 2 Quantitative Genomic Medicine Laboratories (qGenomics), Esplugues del Llobregat, Spain; 3 Hospital Universitario Puerta de Hierro, Madrid, Spain; 4 Barcelona Institute for Global Health (ISGlobal) and Centro de Investigación Biomédica en Red en Epidemiologia Y Salud Pública (CIBERESP), Barcelona, Spain; 5 Universitat Pompeu Fabra (UPF), Barcelona, Spain; 6 Department of Surgery, School of Medicine and Health Sciences and Hospital Clinic de Barcelona (IDIBAPS), University of Barcelona, Calle Sabino de Arana 1 (2nd floor, Ophthalmology), 08028 Barcelona, Spain; 7 Institute of Biomedical Research Sant Pau (IIB-Sant Pau, SGR1113) and Barcelona Tissue Bank (BST), Barcelona, Spain

**Keywords:** Genetic markers, Genetics, Biomarkers, Diseases, Health care, Medical research, Molecular medicine

## Abstract

Predicting the therapeutic response to ocular hypotensive drugs is crucial for the clinical treatment and management of glaucoma. Our aim was to identify a possible genetic contribution to the response to current pharmacological treatments of choice in a white Mediterranean population with primary open-angle glaucoma (POAG) or ocular hypertension (OH). We conducted a prospective, controlled, randomized, partial crossover study that included 151 patients of both genders, aged 18 years and older, diagnosed with and requiring pharmacological treatment for POAG or OH in one or both eyes. We sought to identify copy number variants (CNVs) associated with differences in pharmacological response, using a DNA pooling strategy of carefully phenotyped treatment responders and non-responders, treated for a minimum of 6 weeks with a beta-blocker (timolol maleate) and/or prostaglandin analog (latanoprost). Diurnal intraocular pressure reduction and comparative genome wide CNVs were analyzed. Our finding that copy number alleles of an intronic portion of the *MLIP* gene is a predictor of pharmacological response to beta blockers and prostaglandin analogs could be used as a biomarker to guide first-tier POAG and OH treatment. Our finding improves understanding of the genetic factors modulating pharmacological response in POAG and OH, and represents an important contribution to the establishment of a personalized approach to the treatment of glaucoma.

## Introduction

Glaucoma, a leading cause of irreversible blindness worldwide, is characterized by an optic nerve neuropathy that can develop at any age, with Mendelian inheritance typical for early-onset disease, and complex inheritance typical of adult-onset disease (before and after age 40, respectively)^1^. Primary open-angle glaucoma (POAG), the most prevalent form of the disease, is related to increased intraocular pressure (IOP). Its treatment poses challenges, and interestingly, clinical and therapeutic response can also be influenced by environmental and genetic factors^2,3^. Pharmacological treatment is the main therapeutic pillar for glaucoma, with topical beta-blockers and prostaglandin analogs as the first-line hypotensive drugs used to treat ocular hypertension (OH)^4^. Expected hypotensive capacity, according to several studies, is 20–25% for beta-blockers, and 25–35% for prostaglandins^4–6^. However, the response profile to pharmacological treatment differs in patients. The above-mentioned drugs (in monotherapy or in combination) have also been found to cause intolerance as a result of both topical and systemic undesirable adverse effects^4^.

Pharmacogenomics aims to study the way individual genome variations influence drug response. Genetic variations can affect drug bioavailability, metabolism, sensitivity, toxicity, and dosage. Detailed knowledge of drug pharmacogenetics enables patient management to be optimized by dosage customized according to an individual’s genetic profile^7,8^. The main medical value of this approach is that it is possible to predict, for each individual, whether a drug at a specific dose will be effective or whether it should be avoided due to a high risk of toxicity or lack of response^8,9^. To date, the most common and most extensively studied inter-individual genetic variations that shape pharmacological response are single nucleotide variations together with small insertions/deletions (indels). Other studies have focused on genome structural variations, including inversions, translocations, and copy number variants (CNVs), which have also been found to be a rich source of genetic variability^10–12^. The consequence of structural variation is usually that part of or a whole gene sequence is rearranged (inverted, duplicated, deleted, etc.), commonly resulting in interference with gene functioning. Functionally relevant CNVs have been previously described in genes related to drug absorption, distribution, metabolism, and excretion, including cytochrome P450 (*CYP*) and gluthatione S-trasferase (*GST),* with significantly different frequencies across human populations^8,12,13^. Also of interest are genome-wide association studies (GWAS), as these have successfully identified *loci* contributing to complex diseases and traits^14–16^, including pharmacogenomic response. However, apart from other limitations, the high cost and the need for very large cohorts have hampered the generalization of such approaches.

Recently, various studies have tried to elucidate a genetic basis for drug response to glaucoma treatment. In Chinese patients with glaucoma, for example, a differential response to latanoprost has been described for single-nucleotide polymorphisms (SNPs) in the prostaglandin F2-alpha receptor (*PTGFR*) and solute carrier organic anion transporter family member 2A1 (*SLCO2A1*) genes^17^, with the study concluding that 2 SNPs-rs3766355 in *PTGFR* and rs4241366 in *SLCO2A1*—correlated with a good drug response over both the short- and long-term^17^. More recently, a genotyping study of a cohort of Mediterranean patients with POAG identified 5 SNPs related to response to latanoprost, with *PTGFR* SNPs associated with good (rs6686438, rs10786455) and negative (rs3753380, rs6672484, rs11578155) responses. Interestingly, rs3753380 has previously been reported to be related to a poor response to latanoprost in healthy Japanese subjects^18,19^. The matrix metalloproteinase 1 (*MMP-1*) gene has also been related to refractoriness to latanoprost^20^.

Given the lack of studies exploring the contribution of CNVs to individual pharmacological treatment responses, our aim was to examine the effect of this type of genetic variation in the individual response to latanoprost (prostaglandin) or timolol maleate (beta-blocker) in a white Mediterranean population treated for POAG or OH. We observed that a CNV located in an intronic portion of the muscle-enriched A-type lamin-interacting protein (encoded by the *MLIP* gene) predicts a better response to prostaglandins and, concomitantly, a poorer response to beta-blockers. Analysis of this genetic variant could contribute to better clinical management of glaucoma patients.

## Material and methods

This study followed the ethical precepts of the Declaration of Helsinki (Fortaleza, Brazil, Oct 2013) and was approved by a local ethics committee (CEIC del Centro de Oftalmología Barraquer Barcelona; Ref. COB033). Human samples were obtained, processed, and analyzed in accordance with current EU regulations on the collection and preservation of human tissues and samples (2004/23/CE and 2006/17/CE), and in accordance with the protocol and legal requirements on the use of biological samples for biomedical research in Spain (Law 14/2007 and Royal Decree 1716/2011). The patients received clinical information and gave their informed consent to participate in the study. The use, protection, communication, and transfer of the personal data of participating patients complied with local regulations (Organic Law 5/2018).

### Patients

Included in the study were patients aged 18 and older of both genders, without prior diagnoses and presenting with POAG or OH in one or both eyes requiring pharmacological treatment. IOP was measured using a Goldmann applanation tonometer (Carl Zeiss, Inc., Jena, Germany) mounted on a slit-lamp biomicroscope. IOP measurements were always made at the same time (9 am ± 1 h) by the same researcher (MIC) using the same tonometer, calibrated monthly according to protocol. As diagnostic criteria we considered the following: for POAG, IOP > 21 mmHg associated with optic disc or retinal nerve fiber layer abnormalities, and/or reproducible visual field abnormalities ([4]); and for OH, IOP > 21 mmHg without optic disc or retinal nerve fiber layer abnormalities.

Exclusions from the study were as follows: patients with corneal alterations that could hamper tonometer measurements; patients with pachymetry > 600 µm or < 500 µm; patients with pseudoexfoliative, pigmentary, or secondary glaucoma; patients who had undergone ocular surgery, with the exception of uncomplicated cataract surgery or laser procedures concerning the anterior segment if performed more than 6 months previously; patients with active ocular diseases; and patients currently treated or treated in the previous 6 months with topical, dermatological, intramuscular, or systemic steroids (Table S1, supplementary data).

### Experimental design, cohort, and evaluation

**Figure 1:**
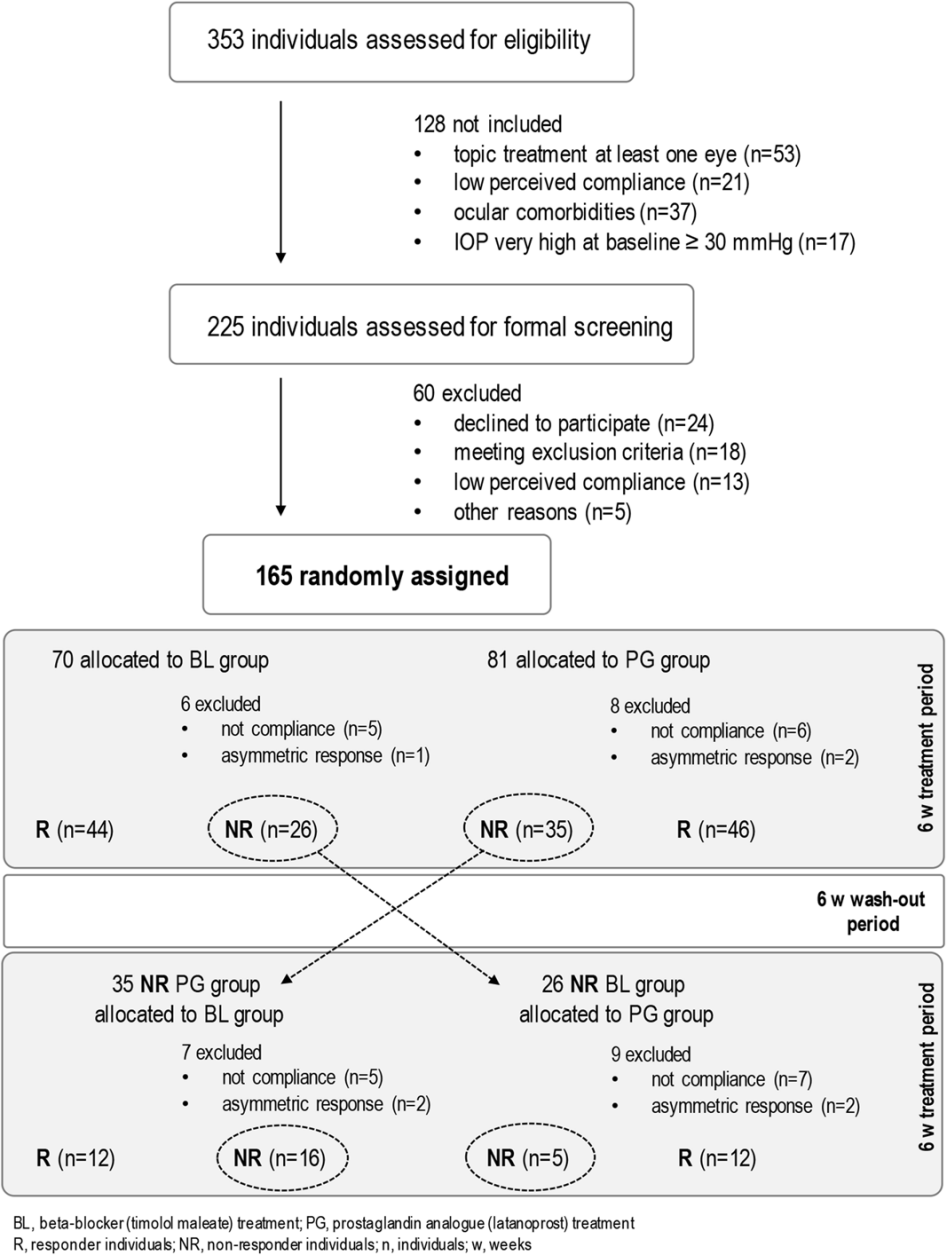
Study flowchart, design and cohort.

A single-center prospective controlled randomized interventional partial crossover study was conducted (Fig. 1). An initial (baseline) tonometry measurement was made before starting treatment, and a second tonometry measurement to evaluate treatment response was made after 6 weeks (42 ± 3 days). According to consensus criteria, the efficacy of baseline IOP reduction attributable to prostaglandin derivatives and to beta-blockers is 25–35% and 20–25%, respectively^4–6^.

Patients were randomized to 2 groups receiving hypotensive treatment as follows. PG group patients received the topical prostaglandin derivative latanoprost (Xalatan, 50 μg/mL) at night, and response and non-response were defined as IOP ≥ 30% and IOP < 25% reduction over baseline, respectively. BL group patients received the topical beta-blocker timolol maleate (Timoftol, 5 mg/mL) every 12 h, and response and non-response were defined as IOP ≥ 25% and IOP < 20% reductions over baseline, respectively^4–6^. After the second tonometry measurement, non-responders were assigned to a wash-out period of 6 weeks and their treatment was crossed over (to timolol maleate for latanoprost non-responders, and to latanoprost for timolol maleate non-responders) for a further 6 weeks, after which they were reassigned to the corresponding group according to response (Fig. 1). Patients with elevated IOP in both eyes, who presented an unsatisfactory therapeutic response in one of them^4^, were not considered in the final analysis.

### DNA samples and genetic analysis

The GWAS analysis of CNVs was conducted in 2 sets of pooled samples meeting the inclusion criteria: for PG group responders and non-responders, and for BL group responders and non-responders. DNA samples were collected at a screening visit before the intervention. Genomic DNA was isolated from 2 mL of saliva using the Oragene collector (ORAGENE-DNA, OG-500; DNA Genotek Inc, Ottawa, Canada), following manufacturer’s recommendations. DNA concentration and purity were measured, using a Denovix DS-11 device (Wilmington, DE, USA), after reading optical density at 260/280 and 260/230 ratios along a continuous wavelength. Equimolar amounts (100 ng each) of the DNA samples were mixed together to form each of the pools. We used 21 and 14 different DNA samples to prepare the PG responder and non-responder pools, respectively. To prepare the BL responder and non-responder pools, 20 and 13 different DNA samples were used, respectively. High-resolution array-based comparative genomic hybridization (aCGH) (Agilent 1 M, AMADID 021,529) was used to hybridize the Cy3-labelled DNA responder pool against the Cy5-labelled non-responder DNA pool. CNV calling was done using the ADM-2 algorithm, with parameters as follows: threshold 6, and 3 consecutive probes with absolute log2ratio values > 0.25. This ensured an approximate average resolution of 9–12 kb for CNV detection (file Table S2, supplementary data).

This microarray-based discovery assay identified 7 regions as having log2ratio values above or below the threshold, reflecting CNVs for responders and non-responders, respectively. A follow-up validation study using a custom multiplex ligation-dependent probe amplification (MLPA) assay was used to genotype the number of copies for each of the 7 *loci* in a larger cohort of patients, following standard procedures^21,22^. The MLPA mix included 4 control probes targeting copy-number neutral and non-variable regions, plus 7 probes targeting the identified variable regions. Control DNA samples were used to normalize values for each probe. In the validation assay with the custom MLPA mix, we analyzed 189 samples from a total of 151 individuals. CNV was estimated from the multimodal distribution of normalized probe heights, which recapitulated the different combinations of copy number alleles and genotypes.

### Statistical analysis

Quantitative data was described in terms of means and standard deviation while qualitative data was described in terms of frequency distribution. The Kolmogorov–Smirnov test was used to determine distribution normality for the measured variables. A 2-tailed Student t-test was run and *p* < 0.05 was considered statistically significant. Those analyses were performed using the Statistical Package for Social Sciences v18.0 (SPSS Inc., Chicago, IL, USA).

Copy-number genotype distributions between responders and non-responders were compared using Fisher’s exact test, in 3 × 2 contingency tables constructed according to the frequency of the different genotypes (AA, AB, and BB). The null hypothesis, rejected if *p* < 0.05, was that allele frequency distributions were no different between the 2 treatment groups. Association analyses between copy numbers for different *loci* and responses to the 2 treatments were performed, using the SNPassoc R package^23^, by assessing interactions between CNV alleles and treatment. The Bonferroni correction was used to identify which CNVs were significantly associated with response to treatment. A generalized linear model was used to compute the probability of interaction between *MLIP* CNV status and treatment.

## Results

A total of 151 patients were studied, 81 and 70 of whom received prostaglandin treatment with latanoprost (PG group) and beta-blocker treatment with timolol maleate (BL group), respectively. All patients were treated for at least 6 weeks. In the PG group, 46 (56.7%) and 35 (43.3%) patients were responders and non-responders, respectively, while the corresponding figures for the BL group were 44 (62.8%) and 26 (37.2%), respectively. Baseline IOP values for the BL and PG groups were 24.3 ± 2.9 mmHg and 24.4 ± 2.2 mmHg, respectively, reduced after treatment to 19.0 ± 3.1 mmHg and 18.9 ± 3.3 mmHg, respectively. Age, sex, and best corrected visual acuity were not response-related for either drug. Table 1 summarizes the demographic characteristics of the study cohort and features of the treatment response.

**Table 1:** Demographic and clinical characteristics of study cohort.

	Total (N)	R (n)	NR (n)	*p*
**BL**	72	44	28	
Age, years (M ± SD)	50.0 ± 15.3	58.2 ± 14.3	52.5 ± 16.5	0.126
Female (%)	39 (54.1%)	26 (59.1%)	13 (46.4%)	0.293
Male (%)	33 (45.9%)	18 (40.9%)	15 (53.6%)	
IOP1, mmHg (M ± SD)	24.3 ± 2.9	24.8 ± 3.2	23.4 ± 2.1	0.063
IOP2, mmHg (M ± SD)	19.0 ± 3.1	17.2 ± 2.3	21.6 ± 2.4	*0.0001*
BCVA (M ± SD)	0.88 ± 0.25	0.92 ± 0.24	0.83 ± 0.26	0.161
**PG**	80	45	35	
Age, years (M ± SD)	57.8 ± 13.4	57.6 ± 13.6	57.9 ± 13.4	0.914
Female (%)	43 (54%)	23 (51%)	20 (57%)	0.447
Male (%)	37 (46%)	22 (49%)	15 (43%)	
IOP1, mmHg (M ± SD)	24.4 ± 2.2	24.6 ± 3.7	24.3 ± 3.1	0.701
IOP2, mmHg (M ± SD)	18.9 ± 3.3	16.8 ± 2.5	21.3 ± 3.1	*0.0001*
BCVA (M ± SD)	0.90 ± 0.29	0.99 ± 0.21	0.80 ± 0.34	*0.006*

BL, beta-blocker (timolol maleate) treatment group; PG, prostaglandin analogue (latanoprost) treatment group; R, responder individuals; NR, non-responder individuals; (N)(n), number of individuals; IOP1, intraocular pressure before treatment; IOP2, after treatment; BCVA, best corrected visual acuity (decimal); M ± SD, mean ± standard deviation; *p,* p-value for responders *vs* non-responders.

Patients not responding to the initial treatment after 6 weeks underwent a wash-out period of 6 weeks, after which treatment was crossed over to the alternative drug (see Fig. 1); these patients were re-evaluated after 6 weeks (42 ± 3 days) according to the established protocol, and their response to the alternative treatment was recorded. This strategy increased the size of the cohort to 189 samples and validated part of the pharmacological response of our cohort: 35 PG non-responders were re-treated with timolol maleate, of whom 12 were reassigned to the BL responder group, 16 were classified as non-responders, and 7 were excluded (not compliance or asymmetric response) from final analysis. In BL group, 26 non-responders were re-treated with latanoprost, of whom 12 were reassigned to the PG responder group, 5 were classified as non-responders, and 9 were excluded (not compliance or asymmetric response). Table 2 summarizes genotype frequencies for *MLIP* copy numbers together with the percentage average response in IOP reduction in our final cohort (i.e., timolol maleate and latanoprost responders and non-responders).

**Table 2:** Percentage average IOP reduction for responder and non-responder genotype frequencies for *MLIP*.

*MLIP*	BL group	PG group	NR group
Total *n* (%)	56 (29.5%)	58 (31.7%)	21 (9.0%)
A/A	2 (23.3%)	9 (33.8%)	1 (0.0%)
A/B	21 (30.5%)	29 (30.2%)	13 (10.2%)
B/B	33 (29.3%)	20 (32.8%)	7 (7.9%)

*MLIP*, muscular LMNA interacting protein; A, 1 copy allele; B, no copies allele; BL, beta-blocker (timolol maleate) treatment group; PG, prostaglandin analogue (latanoprost) treatment group; NR, patients not responsive for any treatment in monotherapy; n, number of individuals; (%), percentage average IOP reduction.

An initial analysis of global CNVs using the high-resolution microarray identified 7 genomic regions (3.8–46 kb in size) – 13q21, glutathione S-transferase theta-1 (*GSTT1*), late cornified envelope 1D *(LCE1D*), phosphatase and actin regulator 1 (*PHACTR1*), *MLIP*, 1p31, and 2q22 – that showed CNVs between the responder and non-responder pooled groups (Table 3 and file Table S2, supplementary data). The custom MLPA validation assay showed that 2 of the 7 candidate loci exhibited significant CNVs between responders and non-responders, namely, 1p31 and *MLIP*. As for the other 5 *loci*, we could not verify significant differences, possibly due to sample selection bias originating in the relatively small number of individuals pooled for the discovery-phase microarray assay.

**Table 3:** Genomic *loci* and differences in copy numbers for responder and non-responder pooled groups (genomic assembly GRCh37).

*Locus*	Chromosome	Cytoband	Start	End	Size (Kb)	*p*
13q21	13	13q21.1	57759370	57788921	29	2001E−23
*GSTT1*	22	22q11.23	24349305	24395353	46	8545E−18
*LCE1D*	1	1q21.3	152762076	152769870	7.7	4322E−07
*PHACTR1*	6	6p24.1	13156200	13159684	3.8	1543E−07
*MLIP*	6	6p12.1	53929240	53934834	5.5	4883E−10
1p31	1	1p31.1	72766555	72801950	35	3764E−31
2q22	2	2q22.3	146865725	146876364	10	263E−12

*GSTT1*, glutathione S-transferase theta 1; *LCE1D*, late cornified envelope 1D; *PHACTR1*, phosphatase and actin regulator 1; *MLIP*, muscular LMNA interacting protein.

**Figure 2:**
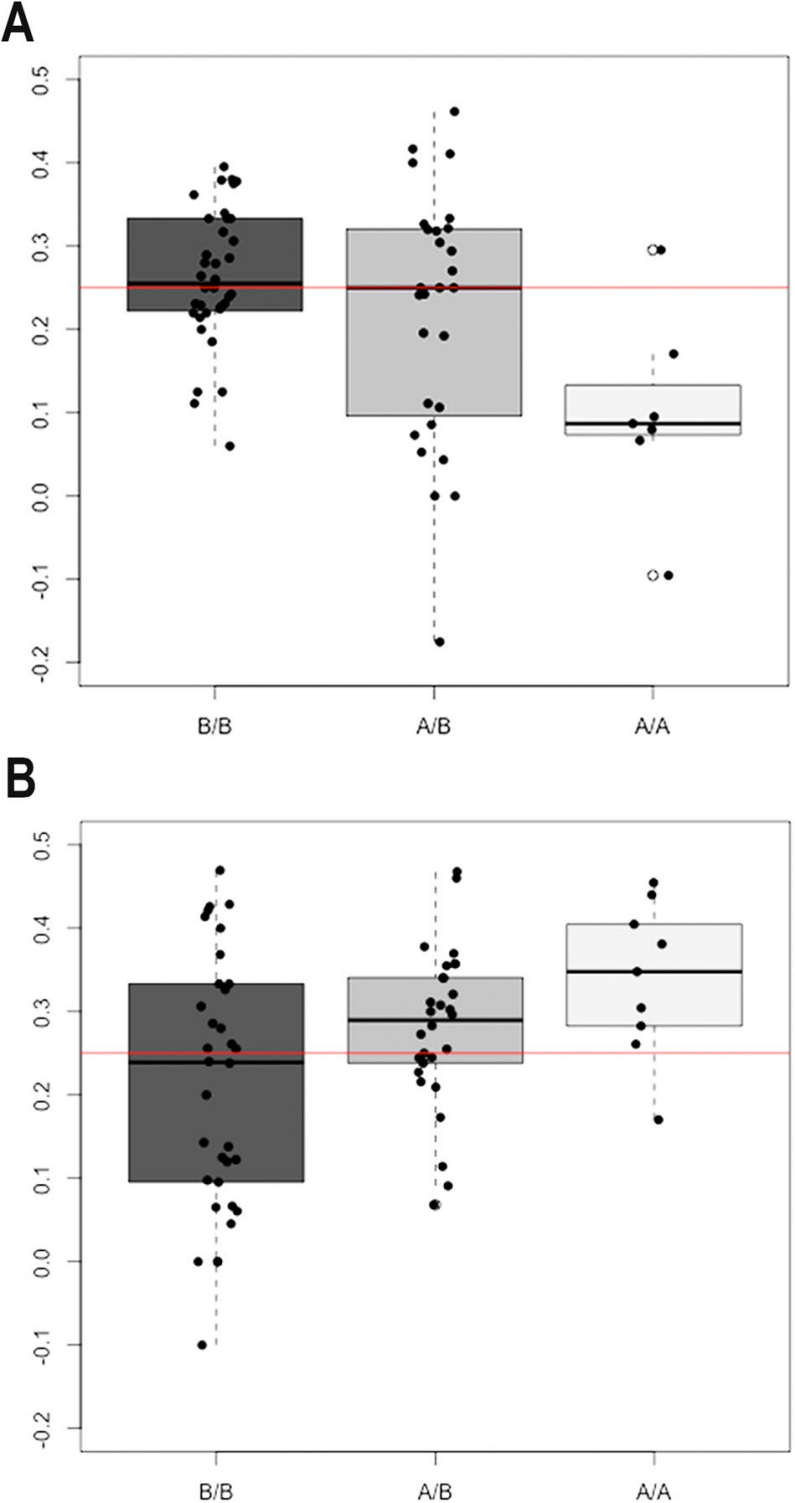
Correlation between copy number of the MLIP *locus* and intraocular pressure (IOP) reduction response to timolol maleate (**A**) and to latanoprost (**B**). Boxplots in each panel shows the percentage distribution of IOP reductions for individuals (black dots) grouped according to their *MLIP* genotype (B: no copy; A: 1 copy). The horizontal red line marks a 25% IOP reduction that differentiates responders from non-responders. Panel A. IOP reduction in response to a beta-blocker (timolol maleate). More copy numbers (AA genotype) tend to be associated with a smaller percentage IOP reduction, while the median response is higher among individuals with 1 or no *MLIP* variant copy. Panel B. Individual responses to prostaglandins (latanoprost) and their genotypes. Individuals with fewer copy numbers show a lower median response to latanoprostf in terms of relative IOP reduction.

For beta-blockers we found more CNVs for non-responders than for responders (Fisher’s exact test; *p* = 0.0039). On further investigation, we found that, under an additive model, each copy of the 1p31 variant allele conferred a negative effect on beta-blocker response in terms of IOP reduction capacity (odds ratio, OR = 0.37; *p* = 0.0009). A similar result was observed for CNVs for the *MLIP locus*, with significant differences in genotype distributions between responders and non-responders (Fisher’s exact test; *p* = 0.02448). Similarly, the more copies of the *MLIP* variant, the weaker the capacity of beta-blockers to reduce IOP (OR = 0.40; *p* = 0.0057) (Fig. 2A).

Interestingly, for the PG group the distribution of *MLIP* copy number alleles was significantly different for responders and non-responders (Fisher’s exact test; *p* = 0.03814), with additional CNVs for *MLIP* producing a significantly greater IOP reduction in PG group responders (OR = 2.34, *p* = 0.01057) (Fig. 2B).

**Figure 3:**
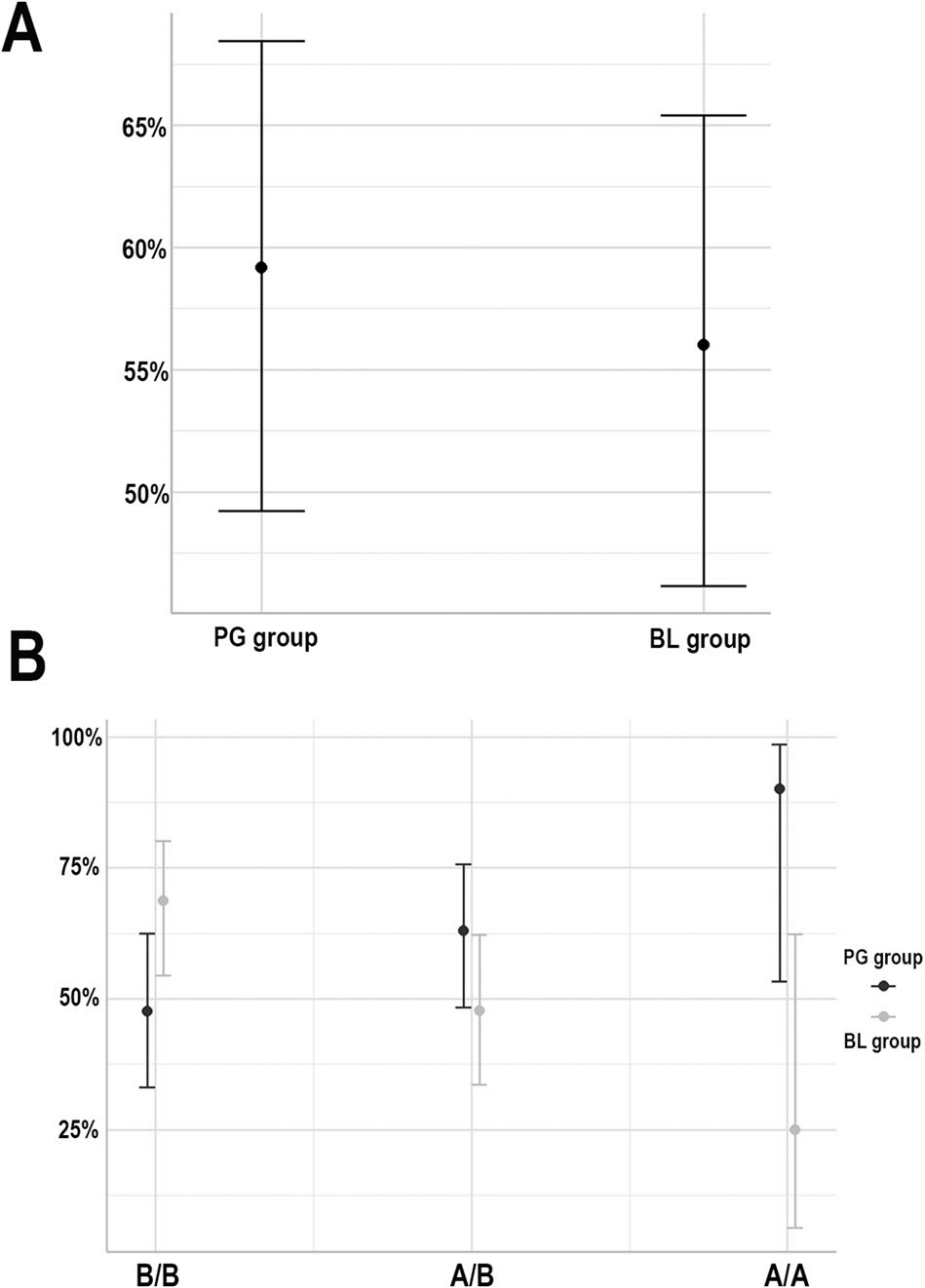
Response probabilities for timolol maleate and latanoprost. (**A**) Overall response probability for both therapeutic groups. (**B**) Response probability related to copy number variant (CNV) genotyping BB (no copy), AB (1 allelic copy), and AA (2 allelic copies) for the *MLIP locus* and the therapeutic groups.

When overall predicted responses were calculated for both the PG and BL groups (Fig. 3A) and related to CNVs for *MLIP* (Fig. 3B), we found response probabilities of almost 90% to latanoprost (PG group) for AA genotypes (2 copies) and of almost 70% to timolol maleate (BL group) for BB genotypes (no copies) (Fig. 3B). However, 21 patients overall (14%) were non-responders to either drug in a monotherapy regimen: 13 AB genotypes (1 copy), 7 BB genotypes (0 copies), and 1 AA genotype (2 copies).

## Discussion

The current concept of personalized medicine means that a patient’s response profile can be anticipated prior to drug treatment, thereby avoiding undesirable systemic and/or local adverse effects, whether mild or severe^24–26^. Genomic analyses have identified key genes contributing to a glaucoma pathophysiology, with glaucoma-related genes defining key biological pathways and processes as potential targets for novel gene-based therapies^1,2^. Personalized glaucoma management relies on a description of genetic variants that impinge on treatment efficacy, minimize pharmacological adverse effects, and ameliorate disease prognosis and progression. Although clinical pharmacogenetic tests are as yet not widely used in ophthalmology, simple and rapid approaches could be applied to therapeutic decision-making.

Our study followed a multi-step approach to identifying and validating the role played by previously unexplored genetic variations in the pharmacogenomic response to two first-line pharmacological treatments for POAG and OH (prostaglandins and beta-blockers). More specifically, we explored the contribution of CNVs, using a strategy based on pooled genomes that aimed to minimize individual CNVs and enrich the common variants present in our group of patients^27–29^. DNA samples from responders and non-responders to prostaglandin and beta-blockers (according to clinical criteria) were independently pooled and analyzed using high-resolution aCGH which identified the 7 *loci* that exhibited copy number differences between responders and non-responders. A custom MLPA assay was used to genotype individual CNV status in a larger cohort of patients.

Our findings illustrate the efficacy of our approach to identifying possible biomarkers of a genetic predisposition to particular pharmacological responses associated with POAG and OH. Our results indicate that initial screening based on studying equimolar DNA pools of carefully phenotyped patients, followed by subsequent individual validation of a larger cohort, is effective.

The validation study showed that pharmacological response was largely influenced by the copy numbers in a 5.6-kb region that overlaps with a known polymorphic CNV^30^, located within intron 1 of the RefSeq *MLIP* gene (accession number NM_138569). Patients higher copy number of *MLIP* variant showed a good response to latanoprost (OR = 2.3), and a poor response to timolol maleate. Conversely, patients with no copies of *MLIP* variant showed a poor response to latanoprost, and a good response to timolol maleate. This interesting finding led us to hypothesize that the two drug types might act through different pathways.

*MLIP* is encoded in the short arm of chromosome 6, and its product interacts with muscular laminin-A (encoded by the *LMNA* gene), a structural component of the nuclear lamina known to be related to familial dilated cardiomyopathy^31–33^. *MLIP* has been proposed as a key regulator of cardiomyopathy and is reported to have a potential as a therapeutic target to attenuate heart failure progression^32,34^. In humans, *MLIP* is strongly expressed in the heart, smooth and skeletal muscle, and brain, weakly expressed in the liver, and not expressed in other human tissues examined to date^32–35^. We were unable to detect mRNA expression for *MLIP* in peripheral blood (data not shown); had we done so, this would have added further value to its potential as a biomarker of therapeutic response.

Interestingly, the region of variability coincides with a recently annotated long non-coding RNA (lncRNA) called MLIP antisense RNA 1 (*MLIP-AS1*). While lncRNAs were initially thought to be transcriptional noise, accumulated and recent evidence points to regulated expression in particular contexts, during both embryonic stem-cell differentiation and adult tissue differentiation^36^. It has also been suggested that lncRNAs are involved in several regulatory processes, including chromatin modification, transcriptional regulation, and post-transcriptional regulation^37^. It therefore cannot be ruled out that absence of this regulation could lead to misregulation of *MLIP* and interfere in drug response at the ocular level. It has also been reported that alternative splicing patterns of *MLIP* mRNA may define versatile and stable regions of *MLIP* likely to modulate its interactions and functions in different tissues^35^; altogether, therefore, further complementary research would be justified to identify its role in eye-related tissues.

As mentioned, previous reports suggest that *MLIP* might play a crucial role in heart disease. While an *MLIP* knock-out mouse model did not show an impact on cardiac function or structure, it did lead to myocardial-specific metabolic abnormalities and cardiac-specific protein kinase B (PKB, or Akt) pathway overactivation, inhibited, in contrast, by cardiac-specific *MLIP* overexpression^32^; this is evidence in favor of a direct impact of *MLIP* on that key signaling pathway. Interestingly, the phosphoinositide-3 kinase (PI3K)/Akt pathway promotes survival and neuroprotection in neurons by inhibiting pro-apoptotic B-cell lymphoma 2 (Bcl-2). Several neuroprotective drugs acting through different mechanisms, including prostaglandin analogs, have been demonstrated to reduce retinal ganglion cell loss and structural damage (caused by increased IOP) through the PI3K/Akt pathway^38^. It is already known that prostaglandin analogs facilitate aqueous humor drainage via uveoscleral flow, as, acting on metalloproteinases and degrading the extracellular matrix, increased uveoscleral outflow in the ciliary muscle reduces IOP^39^. We therefore cannot rule out *MLIP* and Akt signaling pathway participation in IOP response, nor in neuroprotective mechanisms for the optic nerve or different retinal cell populations. Future studies are needed to clarify ciliary muscle and trabecular meshwork protein activation by the PI3K/Akt and Akt/mammalian target of rapamycin (mTOR) signaling pathways in patients with different CNV genotypes for *MLIP*.

To date, no known genes or regulatory elements are affected by the CNV region located in the 1p31 band. The closest gene is human neuronal growth regulator 1 (*NEGR1*), located several kb upstream of the CNVs identified as linked to the response to prostaglandins. *NEGR1* has been associated with a large number of phenotypes in numerous GWAS analyses of complex phenotypes, including body mass index/weight gain, the hemostatic phenotype, immunity, and developmental delay^40,41^. Given the existing evidence, and since a possible physiological effect of these CNVs on *NEGR1* (or other gene) expression has not been conclusively demonstrated, drawing conclusions for a possible relationship with the studied phenotype might be risky.

In conclusion, our findings contribute to knowledge on the genetic factors affecting prostaglandin and beta-blocker response in glaucoma treatment, providing new insights into the genetic architecture and pathways involved in POAG and OH. Nonetheless, since the clinical utility of this finding remains uncertain, high-quality clinical trials are necessary to accumulate sufficient evidence before this knowledge can be transitioned to the clinical setting.

## Supplementary Information

Supplementary Table 1.

Supplementary Table 2.

Supplementary Information.
