# Publisher Correction: MLIP genotype as a predictor of pharmacological response in primary open-angle glaucoma and ocular hypertension

**DOI:** 10.1038/s41598-021-87653-6

**Published:** 2021-04-09

**Authors:** María I. Canut, Olaya Villa, Bachar Kudsieh, Heidi Mattlin, Isabel Banchs, Juan R. González, Lluís Armengol, Ricardo P. Casaroli‑Marano

**Affiliations:** 1 Centro de Oftalmología Barraquer, Instituto Universitario Barraquer (UAB), Barcelona, Spain; 2 Quantitative Genomic Medicine Laboratories (qGenomics), Esplugues del Llobregat, Spain; 3 Hospital Universitario Puerta de Hierro, Madrid, Spain; 4 Barcelona Institute for Global Health (ISGlobal) and Centro de Investigación Biomédica en Red en Epidemiologia Y Salud Pública (CIBERESP), Barcelona, Spain; 5 Universitat Pompeu Fabra (UPF), Barcelona, Spain; 6 Department of Surgery, School of Medicine and Health Sciences and Hospital Clinic de Barcelona (IDIBAPS), University of Barcelona, Calle Sabino de Arana 1 (2nd floor, Ophthalmology), 08028 Barcelona, Spain; 7 Institute of Biomedical Research Sant Pau (IIB-Sant Pau, SGR1113) and Barcelona Tissue Bank (BST), Barcelona, Spain

Correction to: *Scientific Reports* 10.1038/s41598-020-80954-2, published online 15 January 2021

In the original version of this Article, Lluís Armengol was omitted as a corresponding author. Correspondence and request for materials should also be addressed to luis.armengol@qgenomics.com. This error has now been corrected in the HTML and PDF versions of the Article.

