# Supplementary material for: MLIP genotype as a predictor of pharmacological response in primary open-angle glaucoma and ocular hypertension
Source: Sci Rep. 2021 Jan 15;11:1583. doi: 10.1038/s41598-020-80954-2 (PMC7810753; doi:10.1038/s41598-020-80954-2)
Supplement: Supplementary file 1 — Supplementary Table 1. [file 41598_2020_80954_MOESM1_ESM.docx]

**Table S1** (supplementary data). Eligibility cohort criteria.
