# Supplementary material for: MLIP genotype as a predictor of pharmacological response in primary open-angle glaucoma and ocular hypertension
Source: Sci Rep. 2021 Jan 15;11:1583. doi: 10.1038/s41598-020-80954-2 (PMC7810753; doi:10.1038/s41598-020-80954-2)
Supplement: Supplementary file 3 — Supplementary Information. [file 41598_2020_80954_MOESM3_ESM.docx]

**MLIP genotype as a predictor of pharmacological response in primary open-angle glaucoma and ocular hypertension**

**Authors**

María I Canut, MD^1*^

Olaya Villa, PhD^2*^

Bachar Kudsieh, MD PhD^3^

Heidi Mattlin, MSc^2^

Isabel Banchs, MSc^2^

Juan R González, PhD^4,5^

Lluís Armengol, PhD^2,‡^

Ricardo P Casaroli-Marano, MD PhD^6,7,‡^

1. Instituto Universitario Barraquer (UAB), Centro de Oftalmología Barraquer, Barcelona (Spain).

2. Quantitative Genomic Medicine Laboratories (qGenomics), Esplugues del Llobregat (Spain).

3. Hospital Universitario Puerta de Hierro, Madrid (Spain).

4. Barcelona Institute for Global Health (ISGlobal) and Centro de Investigación Biomédica en Red en Epidemiologia y Salud Pública (CIBERESP), Barcelona, Spain.

5. Universitat Pompeu Fabra (UPF), Barcelona, Spain.

6. Department of Surgery, School of Medicine and Health Sciences & Hospital Clinic de Barcelona (IDIBAPS), University of Barcelona, Barcelona (Spain).

7. Institute of Biomedical Research Sant Pau (IIB-Sant Pau, SGR1113) & Barcelona Tissue Bank (BST), Barcelona (Spain).

*MIC and OV contributed equally.

^‡^LA and RPC-M may be considered co-corresponding authors.

**Running head:** Pharmacogenetics in POAG

**Word count:** 3560

**Grants**: No grants to disclose

**Corresponding author:**

Ricardo P Casaroli-Marano, MD PhD

Calle Sabino de Arana 1 (2nd floor, Ophthalmology)

E-08028 – Barcelona (Spain)

Tel: +3493-2275667

E-mail: [rcasaroli@ub.edu](mailto:rcasaroli@ub.edu)

**Financial interest:** Protected by patents 18195545.1 (European Patent Office, 19 Sept 2018) and PCT/ES2019/070606 (13 Sept 2019).
